# Prenatal nicotine exposure enhances Cx43 and Panx1 unopposed channel activity in brain cells of adult offspring mice fed a high-fat/cholesterol diet

**DOI:** 10.3389/fncel.2014.00403

**Published:** 2014-12-02

**Authors:** Juan A. Orellana, Dolores Busso, Gigliola Ramírez, Marlys Campos, Attilio Rigotti, Jaime Eugenín, Rommy von Bernhardi

**Affiliations:** ^1^Departamento de Neurología, Escuela de Medicina, Pontificia Universidad Católica de ChileSantiago, Chile; ^2^Departamento de Nutrición, Diabetes y Metabolismo, Escuela de Medicina, Pontificia Universidad Católica de ChileSantiago, Chile; ^3^Centro de Nutrición Molecular y Enfermedades Crónicas, Escuela de Medicina, Pontificia Universidad Católica de ChileSantiago, Chile; ^4^Laboratorio de Sistemas Neurales, Departamento de Biología, Facultad de Química y Biología, Universidad de Santiago de ChileSantiago, Chile

**Keywords:** hemichannels, connexins, pannexins, nicotine, brain, glia, fat diet

## Abstract

Nicotine, the most important neuroteratogen of tobacco smoke, can reproduce brain and cognitive disturbances *per se* when administered prenatally. However, it is still unknown if paracrine signaling among brain cells participates in prenatal nicotine-induced brain impairment of adult offspring. Paracrine signaling is partly mediated by unopposed channels formed by connexins hemichannels (HCs) and pannexins serving as aqueous pores permeable to ions and small signaling molecules, allowing exchange between the intra- and extracellular milieus. Our aim was to address whether prenatal nicotine exposure changes the activity of those channels in adult mice offspring under control conditions or subjected to a second challenge during young ages: high-fat/cholesterol (HFC) diet. To induce prenatal exposure to nicotine, osmotic minipumps were implanted in CF1 pregnant mice at gestational day 5 to deliver nicotine bitartrate or saline (control) solutions. After weaning, offspring of nicotine-treated or untreated pregnant mice were fed ad libitum with chow or HFC diets for 8 weeks. The functional state of connexin 43 (Cx43) and pannexin 1 (Panx1) unopposed channels was evaluated by dye uptake experiments in hippocampal slices from 11-week-old mice. We found that prenatal nicotine increased the opening of Cx43 HCs in astrocytes, and Panx1 channels in microglia and neurons only if offspring mice were fed with HFC diet. Blockade of inducible nitric oxide synthase (iNOS), cyclooxygenase 2 (COX_2_) and prostaglandin E receptor 1 (EP_1_), ionotropic ATP receptor type 7 (P2X_7_) and NMDA receptors, showed differential inhibition of prenatal nicotine-induced channel opening in glial cells and neurons. Importantly, inhibition of the above mentioned enzymes and receptors, or blockade of Cx43 and Panx1 unopposed channels greatly reduced adenosine triphosphate (ATP) and glutamate release from hippocampal slices of prenatally nicotine-exposed offspring. We propose that unregulated gliotransmitter release through Cx43 and Panx1 unopposed channels may participate in brain alterations observed in offspring of mothers exposed to tobacco smoke during pregnancy.

## Introduction

A growing body of evidence indicates that the risk of developing chronic diseases throughout life is related to environmental factors acting on tissue plasticity at specific windows during fetal development. Maternal cigarette smoking is a well established environmental risk factor associated with adverse effects on fetal outcome, increasing perinatal morbidity and mortality, and evoking long-term behavioral, learning, and memory impairment in the offspring (Naeye and Peters, 1984; Rantakallio and Koiranen, 1987; DiFranza and Lew, 1995; Jacobsen et al., 2006; Julvez et al., 2007). Nicotine is the most important neuroteratogen component of tobacco smoke and, given that it easily crosses the placental and blood–brain barriers (Luck et al., 1985), it is believed to have a dramatic influence on healthy brain development via activation of fetal nicotinic receptors (Dwyer et al., 2008). Indeed, nicotine delivery during pregnancy, eliciting plasma levels comparable to those found in heavy smokers, induces activation of apoptosis-associated genes, long-lasting morphological alterations of neurons, reduced neuronal cell layer thickness, increased number of glial cells, and behavioral impairment (Navarro et al., 1989; Roy and Sabherwal, 1998; Roy et al., 2002; Eugenín et al., 2008). Several studies have attempted to understand the mechanism underlying these nicotine-induced consequences by examining alterations in neurotransmitters (Navarro et al., 1989; Muneoka et al., 1997), changes in nicotinic receptor availability (van de Kamp and Collins, 1994; Coddou et al., 2009), modifications in gene expression (Toledo-Rodriguez et al., 2010; Schneider et al., 2011), and early adaptations of dendritic morphology (Roy and Sabherwal, 1994, 1998). However, the impact of maternal nicotine exposure on the communication of neurons with their partnership cells, the glia, has received little attention.

It is nowadays well established that glial cells express virtually all known neurotransmitter receptors types, allowing them to sense neuronal activity and microenvironmental changes by responding locally through the Ca^2+^-dependent release of bioactive molecules termed “gliotransmitters” (e.g., glutamate, adenosine triphosphate (ATP), adenosine, GABA, and D-serine; Perea et al., 2009; Perea and Araque, 2010). In the central nervous system (CNS), gliotransmitter release is in part mediated by the opening of unopposed membrane channels formed by connexins hemichannels (HCs) or pannexins (Wang et al., 2013b). These channels serve as aqueous pores permeable to ions and small molecules, allowing for diffusional exchange between the intra- and extracellular compartments. In glial cells, HCs and pannexin channels (PCHs) grant the release of gliotransmitters that are necessary for different brain functions including glucosensing (Orellana et al., 2012), ischemic tolerance (Lin et al., 2008), fear memory consolidation (Stehberg et al., 2012), neuron-glia crosstalk (Torres et al., 2012), and chemoreception (Huckstepp et al., 2010). However, several independent studies have pointed out that onset and progression of neurodegenerative homeostatic imbalances may be associated to impairment in permeability properties of these channels in the CNS (Takeuchi et al., 2006; Thompson et al., 2006; Karpuk et al., 2011; Orellana et al., 2011a,b; Gulbransen et al., 2012; Burkovetskaya et al., 2014). Therefore, we decided to investigate whether prenatal nicotine exposure could affect the functional activity of HCs and PCHs in glial cells and neurons in the offspring. Different maternal conditions during pregnancy, including prenatal nicotine, have been shown to sensitize the brain of the adult offspring on its response to a subsequent environmental challenge (Slotkin et al., 1991; Bilbo et al., 2005). Given that fat and cholesterol-enriched diets impair synaptic transmission and glial cell function (Dufour et al., 2006; Triviño et al., 2006; Ya et al., 2013), we studied if dyslipidemia induced by feeding a high-fat/cholesterol (HFC) diet in combination with prenatal nicotine exposure could enhance the opening of connexin and pannexin unopposed channels in the offspring brain. We chose dyslipidemia as a second environmental hit due to the high prevalence of this metabolic condition as a consequence of sedentary lifestyle and overnutrition in Western populations in the last decades.

In this work, we show that prenatal nicotine can increase the opening of unopposed channels formed by connexin 43 (Cx43) in astrocytes and pannexin 1 (Panx1) in microglia and neurons. Interestingly, these responses were only detected when offspring mice were subjected to dyslipidemia induced by feeding them a HFC diet.

## Materials and methods

### Reagents and antibodies

Gap26, Gap19; YGRKKRRQRRRDGANVDMHLKQIEIKKFKYGIEEHGK (TAT-L2) and ^10^panx1 peptides were obtained from Genscript (New Jersey, USA). HEPES, DMEM, DNAse I, poly-L-lysine, LN-6, ns-398, sc-19220, polyclonal anti-Cx43 antibody, 3-(2-carboxypiperazin-4-yl)propyl-1-phosphonic acid (CPP), Brilliant blue G (BBG), oATP, ethidium (Etd) bromide, and probenecid (Prob) were purchased from Sigma-Aldrich (St. Louis, MO, USA). Fetal calf serum (FCS) was obtained from Hyclone (Logan, UT, USA). Penicillin, streptomycin, polyclonal anti-Panx1 antibody (PI488000), goat anti-mouse Alexa Fluor 488 and goat anti-mouse Alexa Fluor 555 were obtained from Invitrogen (Carlsbad, CA, USA). Anti-NeuN monoclonal antibody was obtained from Chemicon (Martinsried/Munich, Germany). Normal goat serum (NGS) was purchased from Zymed (San Francisco, CA, USA). Anti-GFAP monoclonal antibody was purchased from ICN Chemicals, (Irvine, CA). Anti-Cx43 monoclonal antibody was obtained from BD Biosciences (Franklin Lakes, NJ, USA).

### Animal care and use

All animal experimentation was conducted in accordance with the guidelines for care and use of experimental animals of the National Institute of Health (NIH) and local guidance documents generated by the *ad hoc* committee of the Chilean National Commission of Scientific and Technological Research (CONICYT). The procedures and research plan were approved by the Universidad de Santiago Bioethics Committee. CF-1 mice were obtained from the Public Health Institute and housed at the animal facility of the Laboratory of Neural Systems, Universidad de Santiago de Chile. Mice were housed under a 12-h light-darkness condition, with access to *ad libitum* fresh water and food in a temperature (18–26°C)- and humidity (40–70%)-controlled and well ventilated environment.

### Prenatal nicotine exposure and postnatal feeding with HFC diet

Subcutaneous implantation of osmotic minipumps (model 2004, Alzet) was performed in CF1 pregnant mice at gestational day 5 as previously described (Eugenín et al., 2008). In brief, pumps were implanted through an incision made between scapulae, using strict aseptic conditions, under anesthesia with 60–80/20 mg/kg ketamine/xylazine by intraperitoneal (i.p.) injection. Pumps delivered saline (controls) or nicotine bitartrate (60 mg kg^−1^ day^−1^) at a rate of 0.25 µl h^−1^. Recovery from anesthesia was performed under controlled temperature. Dams were maintained in separate cages and daily supervision was done based on the protocol by Morton and Griffiths (Morton and Griffiths, 1985). After weaning, offspring of nicotine-treated or vehicle-treated pregnant mice were fed *ad libitum* with chow or HFC diet (1.25% cholesterol, 15% total fat, and 0.5% cholic acid; Harlan Teklad, USA) for 8 weeks. This dietary condition induced a ~2–3 fold increase in total plasma cholesterol with no significant effect in body weight (not shown).

### Acute hippocampal slices

Eleven-week-old offspring mice were decapitated, and their brains were dissected and placed in ice-cold artificial cerebrospinal fluid (ACSF) containing (in mM): 125 NaCl, 2.5 KCl, 25 glucose, 25 NaHCO_3_, 1.25 NaH_2_PO_4_, 2 CaCl_2_, and 1 MgCl_2_, bubbled with 95% O_2_/5% CO_2_, pH 7.4. Hippocampal coronal brain slices (400 µm) were cut using a vibratome (Leica, VT 1000GS; Leica, Wetzlar, Germany) filled with ice-cold ACSF. The slices were transferred at room temperature (20–22°C) to a holding chamber and immersed in oxygenated ACSF, pH 7.4, for a stabilization period of 30 min before being used.

### Dye uptake and confocal microscopy

For “snapshot” experiments, acute slices were incubated with 100 µM Etd for 15 min in a chamber with oxygenated (95% O_2_ and 5% CO_2_) ACSF, pH 7.4. Then, they were washed five times with ACSF, fixed at room temperature with 4% paraformaldehyde for 30 min, rinsed extensively in phosphate buffered saline (PBS) and stored overnight at 4°C with cryoprotectant (30% sucrose). Next day, slices were frozen and cut into 12–16 µm-thick cryosections with a cryostat. Cryosections were incubated in 0.1% PBS-Triton X-100 containing 10% NGS for 30 min. Afterwards, they were incubated overnight at 4°C with anti-Iba-1 polyclonal antibody (1:300, Wako), anti-GFAP monoclonal antibody (1:300, Sigma), anti-NeuN monoclonal antibody (1:400, Chemicon), polyclonal anti-Cx43 (1:600, Sigma) or polyclonal anti-Panx1 (1:600, Invitrogen) diluted in 0.1% PBS-Triton X-100 with 2% NGS. After five rinses in 0.1% PBS-Triton X-100, cryosections were incubated with goat anti-rabbit Alexa Fluor 488 (1:1500), goat anti-mouse Alexa Fluor 488 (1:1500) or goat anti-mouse Alexa Fluor 647 (1:1500) at room temperature for 1 h. After several washes, coverslips were mounted in Fluoromount and examined in a confocal laser-scanning microscope (Olympus Fluoview FV1000, Tokyo, Japan). Stacks of consecutive confocal images taken with a 63 X objective at 500 nm intervals were sequentially acquired with two lasers (argon 488 nm and helium/neon 543 nm), and Z projections were reconstructed using Fluoview software. The dye uptake ratio was calculated as the subtraction (F-F0) between the fluorescence (F) from respective cell and the background fluorescence (F0). At least six cells by field were selected from at least three fields in each hippocampal slice. Gap26, Gap19; TAT-L2, ^10^panx1, probenecid, L-N6, ns-398, sc-19220, CPP, BBG and oATP were pre-incubated 15 min and then coapplied with Etd for “snapshot” experiments.

### Measurement of extracellular ATP and glutamate concentration

Acute hippocampal slices were immersed in oxygenated ACSF, pH 7.4, at room temperature (20–22°C) for 30 min. Then, ATP and glutamate concentration in the extracellular solution were measured using a luciferin/luciferase bioluminescence and glutamate assay kit (Sigma-Aldrich), respectively. The amount of ATP and glutamate in each sample were calculated from standard curves and normalized by the protein concentration. Briefly, after the experiments, slices were washed twice with ACSF solution and sonicated in ice-cold PBS containing 5 µM ethylenediaminetetraacetic acid (EDTA), Halt (78440) and T-PER protein extraction cocktail (78510), according to manufacturer instructions (Pierce, Rockford, IL). Proteins were measured using the Bio-Rad protein assay. Gap 26, Gap 19; TAT-L2, ^10^panx1, probenecid, LN-6, ns-398, sc-19220, CPP, BBG and oATP were pre-incubated 30 min before the measurements.

### IL-1β and TNF-α assay

IL-1β and TNF-α were determined in 100 µL of sera. Samples were centrifuged at 14,000 g for 40 min. Supernatants were collected and protein content assayed by the BCA method. IL-1β and TNF-α levels were determined by sandwich ELISA, according to the manufacturer’s protocol (eBioscience, San Diego, CA, USA). For the assay, 100 µl of samples were added per ELISA plate well and incubated at 4°C overnight. A calibration curve with recombinant cytokine was included. Detection antibody was incubated at room temperature for 1 h and the reaction developed with avidin–HRP and substrate solution. Absorbance was measured at 450 nm with reference to 570 nm with the microplate reader Synergy HT (Biotek Instruments).

### Data analysis and statistics

For each data group, results were expressed as mean ± standard error (SEM); n refers to the number of independent experiments. For statistical analysis, each treatment was compared with its corresponding control, and significance was determined using a one-way ANOVA followed, in case of significance, by a Tukey *post hoc* test.

## Results

### Prenatal nicotine enhances Cx43 and Panx1 unopposed channel activity in brain cells of offspring mice fed a high-fat/cholesterol diet

**Figure 1:**
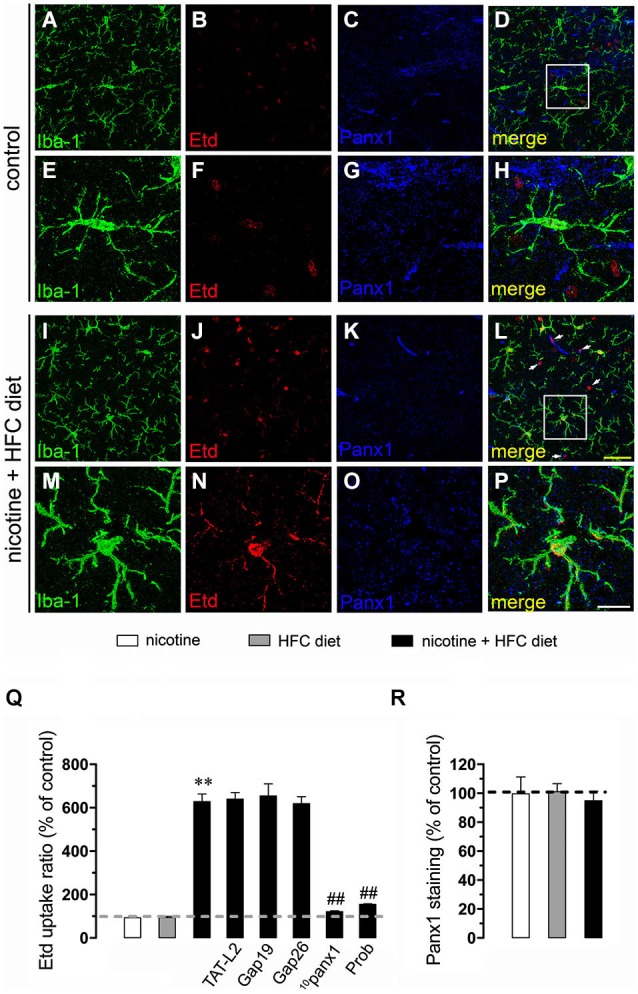
**Exposure to prenatal nicotine and postnatal HFC diet increased Panx1 channel activity in microglia**. Representative images showing Iba-1 (green), Etd (red) uptake and Panx1 (blue) staining of acute hippocampal brain slices made from control mice **(A–H)** or subjected to prenatal nicotine and postnatal HFC diet **(I–P)**. Images of microglia displayed on the bottom insets **E–H** and **M–P** were taken from the zone depicted by the white square in respective panels **D** and **L**. Calibration bars: yellow = 30 µm and white = 10 µm. **(Q)** Averaged data of Etd uptake ratio normalized by the control condition (dashed line) of microglia from mice subjected to prenatal nicotine (white bars), postnatal cholesterol-enriched diet (gray bars), or a combination of both (black bars). Also shown are the effects of the following blockers applied during Etd uptake recordings: TAT-L2 (100 µM), Gap19 (100 µM), Gap26 (100 µM), ^10^panx1 (100 µM) or probenecid (Prob, 500 uM). **(R)** Averaged data of Panx1 staining normalized to control conditions (dashed line) of microglia from mice exposed to prenatal nicotine (white bars), postnatal HFC diet (gray bars), or a combination of both (black bars). **p < 0.005, effect of prenatal nicotine and postnatal HFC diet compared with control conditions. ^##^*p* < 0.005, effect of each blocker compared with the respective effect induced by prenatal nicotine and postnatal HFC diet. Averaged data were obtained from at least three independent experiments.

**Figure 2:**
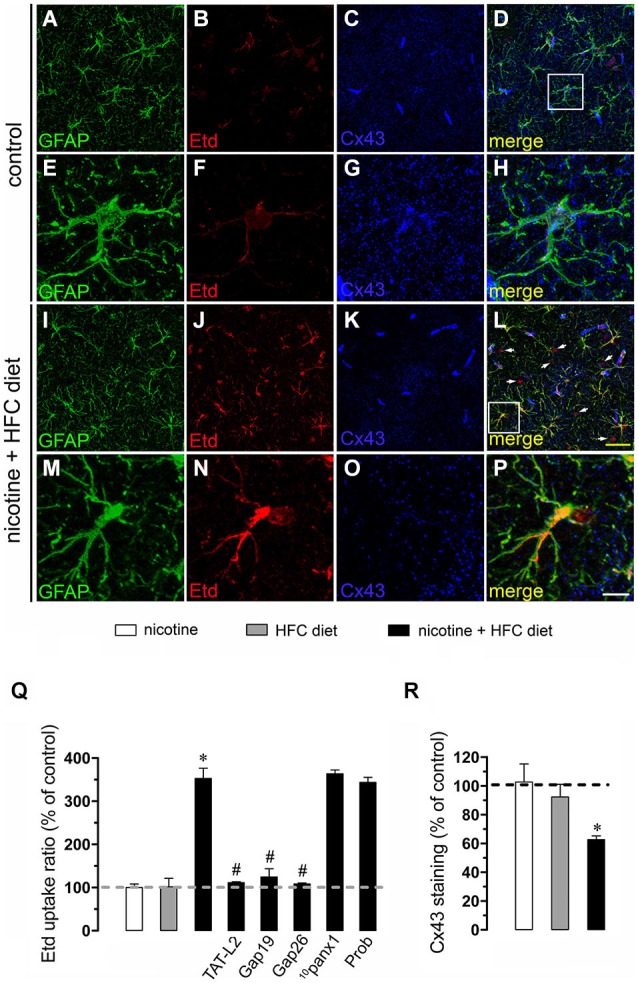
**Exposure to prenatal nicotine and postnatal HFC diet increased Cx43 HC activity in astrocytes**. Representative images showing GFAP (green), Etd (red) uptake and Cx43 (blue) staining of acute hippocampal brain slices made from control mice **(A–H)** or subjected to prenatal nicotine and postnatal HFC diet **(I–P)**. Images of hippocampal astrocytes displayed on the bottom insets **E–H** and **M–P** were taken from the zone depicted by the white square in respective panels **D** and **L**. Calibration bars: yellow = 70 µm and white = 20 µm **(Q)** Averaged data of Etd uptake ratio normalized to control conditions (dashed line) of astrocytes from mice exposed to prenatal nicotine (white bars), postnatal HFC diet (gray bars), or a combination of both (black bars). Also shown are the effects of the following blockers applied during Etd uptake recordings: TAT-L2 (100 µM), Gap19 (100 µM), Gap26 (100 µM), ^10^panx1 (100 µM) or probenecid (Prob, 500 uM). **(R)** Averaged data of Cx43 staining normalized to control conditions (dashed line) of astrocytes from mice exposed to prenatal nicotine (white bars), postnatal HFC diet (gray bars), or a combination of both (black bars). *p < 0.05, effect of prenatal nicotine and postnatal HFC diet compared with control conditions. ^#^*p* < 0.05, effect of each blocker compared with the effect induced by prenatal nicotine plus a postnatal HFC diet. Averaged data were obtained from at least three independent experiments.

**Figure 3:**
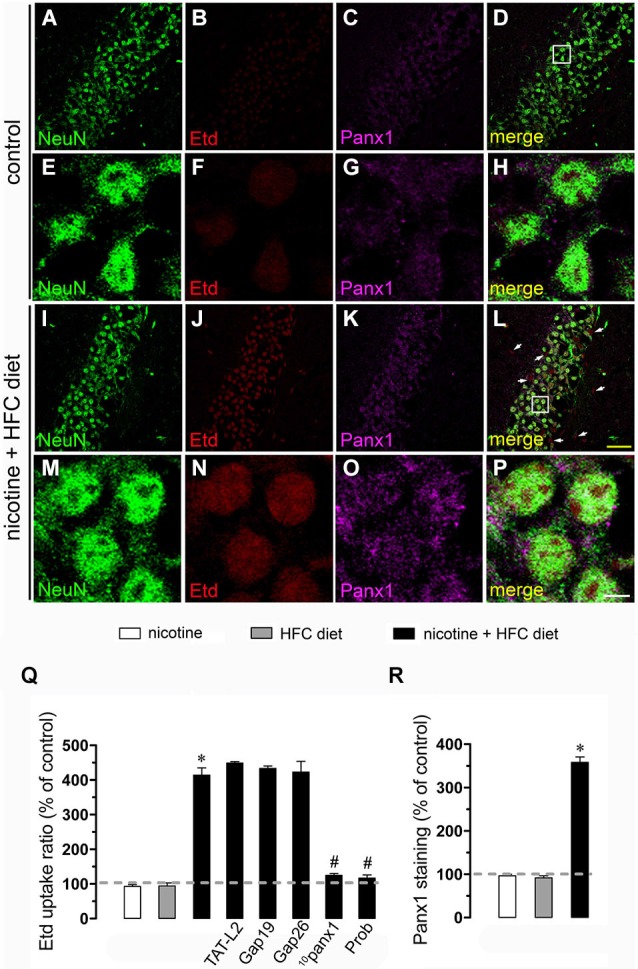
**Exposure to prenatal nicotine and postnatal HFC diet increased Panx1 channel activity in neurons**. Representative images showing NeuN (green), Etd (red) uptake and Panx1 (magenta) staining of acute hippocampal brain slices made from control mice **(A–H)** or subjected to prenatal nicotine and postnatal HFC diet **(I–P)**. Images of hippocampal neurons displayed on the bottom insets **E–H** and **M–P** were taken from the zone depicted by the white square in respective panels **C** and **I**. Calibration bars: yellow = 90 µm and white = 10 µm. **(Q)** Averaged data of Etd uptake ratio normalized with control conditions (dashed line) of neurons from mice subjected to prenatal nicotine (white bars), postnatal HFC diet (gray bars), or a combination of both (black bars). Also shown are the effect of the following blockers applied during Etd uptake recordings: TAT-L2 (100 µM), Gap19 (100 µM), Gap26 (100 µM), ^10^panx1 (100 µM) or probenecid (Prob, 500 uM). **(R)** Averaged data of Panx1 staining normalized with control conditions (dashed line) of neurons from mice subjected to prenatal nicotine (white bars), postnatal HFC diet (gray bars), or a combination of both (black bars). *p < 0.05, effect of prenatal nicotine and postnatal HFC diet compared with control conditions. ^#^*p* < 0.05, effect of each blocker compared with the effect induced by prenatal nicotine plus a postnatal HFC diet. Averaged data were obtained from at least three independent experiments.

Nicotine delivery during pregnancy induces neuronal alterations and behavioral impairment (Navarro et al., 1989; Roy and Sabherwal, 1998; Roy et al., 2002). On the other hand, the opening of HCs and PCHs has been linked to glial cell dysfunction and neuronal impairment (Takeuchi et al., 2006; Orellana et al., 2011a,b, 2013; Shestopalov and Slepak, 2014). Therefore, we investigated whether prenatal nicotine exposure could affect the functional activity of these channels in microglia, astrocytes and neurons of the offspring. To address this, we examined HC and PCH activity by measuring Etd uptake in acute hippocampal slices from 11-week-old offspring. Etd is a molecule that crosses the plasma membrane in healthy cells by passing through specific large channels, including connexin and pannexin unopposed channels. Upon binding to intracellular nucleic acids, Etd becomes fluorescent, indicating channel opening when appropriate blockers are employed (Schalper et al., 2008; Sáez and Leybaert, 2014). Etd uptake by Iba-1-positive microglia, GFAP-positive astrocytes and NeuN-positive neurons on acute hippocampal slices was evaluated in “snapshot” experiments. Microglia, astrocytes and neurons from offspring of control dams showed a low Etd uptake ratio (Figures 1A–H, 2A–H, 3A–H) as previously reported (Orellana et al., 2010; Karpuk et al., 2011). Interestingly, prenatal exposure to nicotine did not change Etd uptake in brain cells in the offspring compared to control conditions (Figures 1Q, 2Q, 3Q).

Further, we evaluated whether prenatal nicotine sensitized the brain of adult offspring on its response to a HFC diet. Similarly to what was observed in the offspring from control dams fed a chow diet, Etd uptake remained low when the offspring from control dams was fed a HFC diet (Figures 1Q, 2Q, 3Q). However, HFC diet increased Etd uptake in microglia, astrocytes and pyramidal neurons when the offspring came from nicotine-treated dams (Figures 1I–Q, 2I–Q, 3I–Q). Microglia express functional unopposed channels formed by Panx1 and Cx43 (Orellana et al., 2011b). The possible role of Panx1 channels in nicotine-evoked Etd uptake was studied using probenecid and the mimetic peptide ^10^panx1 with an amino acid sequence homologous to the second loop of Panx1 (Pelegrin and Surprenant, 2006; Silverman et al., 2008). Probenecid (500 µM) and ^10^panx1 (200 µM) nearly abolished the increased microglial cell Etd uptake triggered by prenatal nicotine and postnatal HFC diet (Figures 1I–Q). In contrast, mimetic peptides homologous to the cytoplasmic (TAT-L2 and Gap19; 100 µM) or first extracellular (Gap26; 100 µM) loop of Cx43 (Wang et al., 2013a), did not reduce nicotine-induced Etd uptake by microglia (Figure 1Q).

Astrocytes express functional unopposed channels formed by Cx43 (Contreras et al., 2002) and Panx1 (Iglesias et al., 2009). Thereby, we used TAT-L2, Gap19, Gap26, probenecid and ^10^panx1 to determine the contribution of both channels in the nicotine-induced Etd uptake by astrocytes. TAT-L2 (100 µM), Gap19 (100 µM) and Gap26 (100 µM) fully reduced astroglial cell Etd uptake evoked by prenatal nicotine and postnatal HFC diet (Figure 2Q). In contrast, ^10^panx1 (100 µM) and probenecid (500 µM) failed to induce the same inhibition (Figure 2Q).

For neurons, most evidence support that they express unopposed channels formed by Panx1 (Thompson et al., 2006). In agreement with that evidence, ^10^panx1 and probenecid strongly reduced the nicotine and postnatal HFC diet-induced Etd uptake observed in pyramidal neurons (Figure 3Q), whereas TAT-L2, Gap19 and Gap26 failed to cause a similar response (Figure 3Q). Overall, these data support the idea that prenatal nicotine plus postnatal HFC diet increases the opening of unopposed channels formed by Cx43 in astrocytes and Panx1 in microglia and neurons.

### Prenatal nicotine affects levels of Cx43 and Panx1 in brain cells of offspring mice fed a high-fat/cholesterol diet

Given that pathological conditions affect the expression of connexins and pannexins in the CNS (Rouach et al., 2002; Orellana et al., 2009), we examined whether prenatal nicotine plus postnatal HFC diet could modulate Cx43 and Panx1 levels in brain cells by confocal analysis. As expected, neither prenatal nicotine nor postnatal HFC diet alone affected Cx43 and Panx1 levels in astrocytes and neurons, respectively (Figures 2R, 3R). However, combination of prenatal nicotine plus feeding a HFC diet during adulthood reduced Cx43 levels in astrocytes (Figure 2R), whereas in pyramidal neurons, immunodetection of Panx1 was increased (Figure 3R). For all tested conditions, Panx1 remained unchanged in microglia (Figure 1R).

### Increased opening of Cx43 and Panx1 unopposed channels in nicotine and high-fat/cholesterol exposed offspring brain depends on iNOS/COX2/EP1 receptor pathway and purinergic/glutamatergic signaling

**Figure 4:**
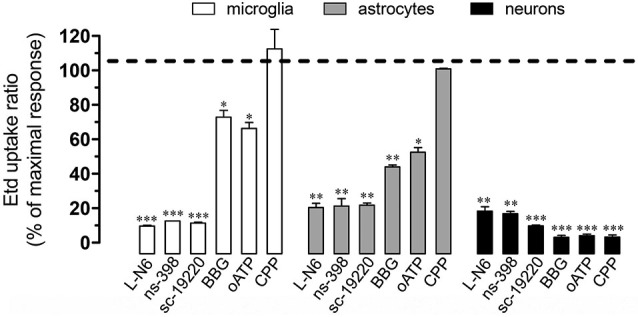
**Cx43 and Panx1 unopposed channel activity evoked by prenatal nicotine and postnatal HFC diet depended on the iNOS/COX_2_/EP_1_ receptor pathway and NMDA/P2X_7_ receptor signaling**. Averaged data normalized to maximal Etd uptake ratio (dashed line) achieved by prenatal nicotine and postnatal HFC diet in microglia (white bars), astrocytes (gray bars) and neurons (black bars) treated with the following blockers during Etd uptake recordings: L-N6 (1 µM), ns-398 (5 µM), sc-19220 (20 µM), BBG (200 µM), oATP (10 µM) and CPP (20 µM). * *p* < 0.05, ** *p* < 0.005; *** *p* < 0.001, effect of each blocker compared with the effect induced by prenatal nicotine plus a postnatal HFC diet. Averaged data were obtained from at least three independent experiments.

Under neuroinflammatory conditions, glial cells exhibit a prominent activation of inducible nitric oxide (NO) synthase (iNOS) and cyclooxygenase 2 (COX_2_; Tzeng et al., 2005; Amitai, 2010), two enzymes that produce mediators (NO and prostaglandins, respectively) linked to the opening of Cx43 and Panx1 unopposed channels (Retamal et al., 2007; Orellana et al., 2013). Accordingly, we investigated the contribution of iNOS and COX_2_ activation on Etd uptake induced by prenatal nicotine and postnatal HFC diet. Notably, iNOS and COX_2_ inhibition by L-N6 (1 µM) and ns-398 (5 µM), respectively, greatly reduced Etd uptake induced by prenatal nicotine and postnatal HFC diet in microglia, astrocytes and pyramidal neurons (Figure 4). It has been previously shown that iNOS-dependent release of NO increases COX_2_ activity and prostaglandin E_2_ (PEG_2_) production in macrophages (Salvemini et al., 1993). Importantly, PEG_2_ activates G protein-coupled receptor 1 (EP_1_), increasing the intracellular free Ca^2+^ concentration ([Ca^2+^]_*i*_; Woodward et al., 2011), causing opening of Panx1 unopposed channels (Orellana et al., 2013). Thus, we assessed whether the EP_1_ receptor participated in the above mentioned responses. Inhibition of the EP_1_ receptor with sc-19220 (20 µM) resulted in a prominent reduction of the Etd uptake triggered by prenatal nicotine and postnatal HFC diet in microglia, astrocytes and pyramidal neurons (Figure 4).

When activated, glial cells release relevant amounts of gliotransmitters including ATP and glutamate, which underlie glia-to-glia communication via activation of purinergic and glutamatergic receptors (Perea et al., 2009; Perea and Araque, 2010). Because opening of HCs and PCHs has been asociated with purinergic and glutamatergic signaling (Locovei et al., 2006; Thompson et al., 2008; Orellana et al., 2011a,b), we examined if NDMA and ionotropic ATP receptor type 7 (P2X_7_) receptors were involved in the Etd uptake induced by prenatal nicotine and postnatal HFC diet. Remarkably, BBG (10 µM) and oATP (200 µM), two blockers of P2X_7_ receptors, partially reduced the nicotine-induced Etd uptake in microglia and astrocytes, whereas it achieved an almost complete inhibition on pyramidal neurons (Figure 4). In addition, the NMDA receptor blocker CPP (20 µM), completely abolished Etd uptake evoked by prenatal nicotine and postnatal HFC diet in pyramidal neurons, whereas it failed to show the same inhibitory effect in glial cells (Figure 4). Taken together, these data indicate that the increase in Etd uptake induced by prenatal nicotine and postnatal HFC diet depended on activation of the iNOS/COX_2_/EP_1_ receptor pathway and signaling via P2X_7_/NMDA receptors.

### Prenatal nicotine induces Cx43 and Panx1-dependent release of ATP and glutamate in brain cells of offspring mice fed a high-fat/cholesterol diet

**Figure 5:**
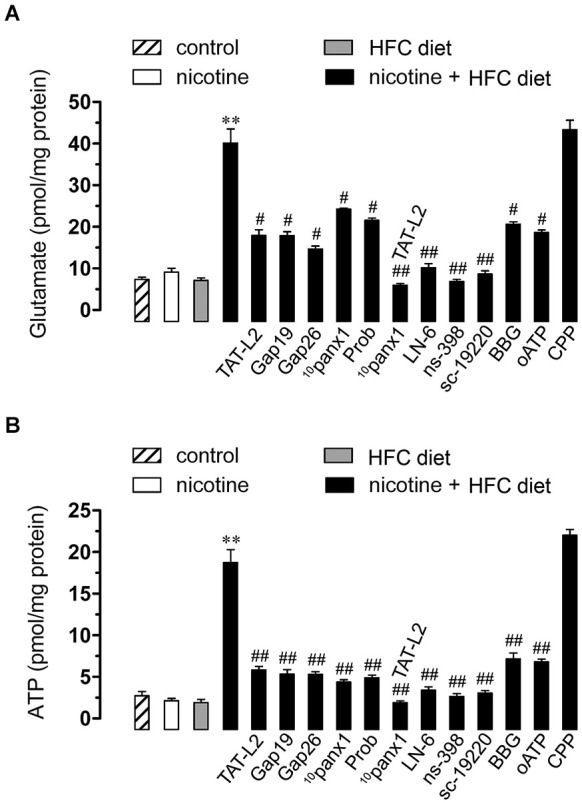
**Opening of Cx43 and Panx1 unopposed channels allowed for the release of glutamate and ATP via the iNOS/COX_2_/EP_1_ receptor pathway and NMDA/P2X_7_ receptor signaling**. Averaged data of glutamate **(A)** or ATP **(B)** release by hippocampal slices made from mice maintained under control conditions (dashed bar), or exposed to prenatal nicotine (white bar), postnatal HFC diet (gray bars) or a combination of both (black bars). Also shown are the effect of the following blockers applied during the experiments: TAT-L2 (100 µM), Gap19 (100 µM), Gap26 (100 µM), ^10^panx1 (100 µM), probenecid (Prob, 500 uM), L-N6, ns-398, sc-19220, BBG, oATP or CPP. ** *p* < 0.005, effect of prenatal nicotine plus postnatal HFC diet compared with control conditions. ^#^ *p* < 0.05, ^##^ *p* < 0.005; effect of each blocker compared with the effect induced by prenatal nicotine plus a postnatal HFC diet. Averaged data were obtained from at least three independent experiments.

Recently, it has been demonstrated that gliotransmitters elicit their own release in an autocrine manner via Cx43 and Panx1 unopposed channels (Orellana et al., 2012, 2013). Given that NMDA/P2X_7_ receptors were involved in the Etd uptake observed in the offspring exposed to prenatal nicotine and fed a HFC diet during adulthood, we next evaluated whether glutamate and ATP release from hippocampal slices via Cx43 and/or Panx1 unopposed channels were also affected in this condition. Similarly to that observed in Etd uptake experiments, neither prenatal nicotine nor postnatal HFC diet by themselves affected the release of both gliotransmitters compared with control conditions (Figures 5A,B). However, the exposure to nicotine prenatally combined with a HFC diet during adult life strongly increased the release of glutamate and ATP (Figures 5A,B). Interestingly, TAT-L2 (100 µM), Gap19 (100 µM) and Gap26 (100 µM) prominently reduced the release of glutamate and ATP induced by prenatal nicotine and postnatal HFC diet (Figures 5A,B). Similar effects were observed on the release of glutamate and ATP upon treatment with ^10^panx1 and probenecid (Figures 5A,B). Taken together, these results indicate that exposure to prenatal nicotine plus postnatal HFC diet increased the release of glutamate and ATP by the opening of unopposed channels formed by Cx43 and Panx1.

As expected, L-N6 (1 µM), ns-398 (5µM) and sc-19220 (20 µM) fully inhibited the release of glutamate and ATP triggered by prenatal nicotine exposure and HFC diet (Figures 5A,B). Furthermore, supporting the idea that gliotransmitters can elicit their own release, we found that BBG (10 µM) and oATP (200 µM) almost completely abolished the release of glutamate and ATP induced by prenatal nicotine and postnatal HFC diet. However, blockade of NMDA receptors with CPP did not show the same effect (Figures 5A,B). The evidence suggest that ATP but not glutamate, could partially evoke its own release by an autocrine pathway possibly mediated by Cx43 and Panx1 unopposed channels.

### Prenatal nicotine and postnatal high-fat/cholesterol diet increased serum levels of IL-1β

**Figure 6:**
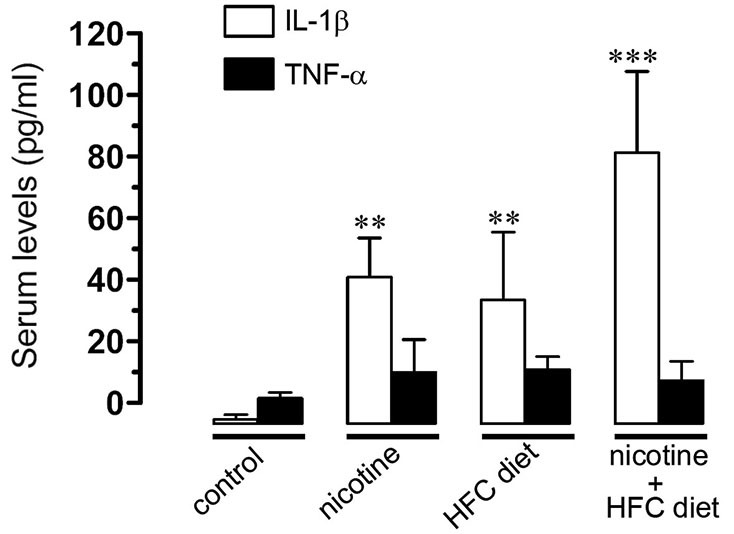
**Prenatal nicotine and postnatal HFC diet increased serum levels of IL-1beta**. Averaged data of serum levels of IL-1β (white bars) and TNF-α (black bars) from mice under control conditions, or subjected to prenatal nicotine, postnatal HFC diet, or a combination of both conditions. ** *p* < 0.005, *** *p* < 0.001; effect of treatments compared with control conditions. Averaged data were obtained from at least five animals.

Given that previous studies have shown that IL-1β and TNF-α increase the opening of HCs and PCHs in glial cells (Retamal et al., 2007; Froger et al., 2009, 2010; Sáez et al., 2013), we evaluated serum levels of IL-1β and TNF-α in the offspring. TNF-α levels remained unchanged at the various conditions. However, IL-1β was notably increased by prenatal nicotine or postnatal HFC diet alone, and by the combination of prenatal nicotine plus postnatal HFC diet, being the latest the condition achieving the most robust increase (Figure 6). This evidence indicates that opening of Cx43 and Panx1 unopposed channels evoked by prenatal nicotine exposure and postnatal HFC diet occurred concomitantly with an increased pro-inflammatory state of the offspring.

## Discussion

In this study, we showed that prenatal nicotine and postnatal HFC diet for 8 weeks after weaning increased the opening of unopposed channels formed by Cx43 in astrocytes and Panx1 in microglia and neurons. This enhanced opening occurred by a mechanism depending on iNOS/COX_2_/EP_1_ receptor pathway activation and signaling via P2X_7_/NMDA receptors. In addition, unopposed channel opening resulted in the release of two major gliotransmitters: glutamate and ATP.

Previous studies have demonstrated that nicotine delivery during pregnancy induces neuronal defects, increased number of glial cells, and behavioral impairment in the offspring (Navarro et al., 1989; Roy and Sabherwal, 1998; Roy et al., 2002). Our results suggest that the effect induced by prenatal nicotine could be mediated in part by enhanced release of gliotransmitters. It has been shown that gliotransmitter release through HCs and PCHs underlies crucial functions in the physiology of the CNS (Lin et al., 2008; Huckstepp et al., 2010; Orellana et al., 2012; Stehberg et al., 2012; Torres et al., 2012). Moreover, several studies indicate that uncontrolled opening of these channels results in exacerbated release of gliotransmitters, which in high concentrations can be toxic for neighboring cells (Takeuchi et al., 2006; Orellana et al., 2011a,b). Here, we found that prenatal nicotine in combination with a postnatal HFC diet increased the opening of HCs and PHCs in brain cells. In agreement with their sensor role in the CNS (Block et al., 2007), microglia showed the highest Etd uptake compared with astrocytes and neurons in mice exposed to both environmental stressors. As this response was nearly abolished by Panx1 but not Cx43 unopposed channel blockers, the former protein appeared to be the major responsible for the increased permeability. In agreement with our data, recent studies have shown that pro-inflammatory conditions increase the opening of Panx1 channels in microglia (Orellana et al., 2013; Sáez et al., 2013). Both astrocytes and neurons exhibited similar increases on Etd uptake after prenatal nicotine and postnatal HFC diet. However, this effect was due to Cx43 HCs in the former, as mimetic peptides known to block these channels (Wang et al., 2013a), completely inhibited astroglial cell Etd uptake. In contrast, ^10^panx1 and probenecid did not affect astroglial cell Etd uptake. On the other hand, neuronal Etd uptake induced by prenatal nicotine and postnatal HFC diet was drastically blocked by ^10^panx1 and probenecid but not by TAT-L2, Gap19 or Gap26, indicating that Panx1 channels were the main contributors for this response.

**Figure 7:**
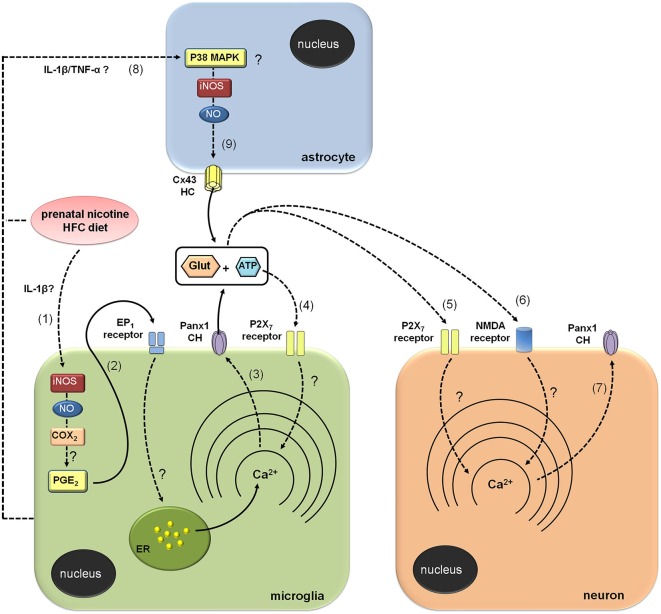
**Modulation of brain cell hemichannels by prenatal nicotine and postnatal high-fat/cholesterol diet**. Prenatal nicotine and postnatal high-fat/cholesterol diet increase serum levels of IL-1β, leading to iNOS/COX_2_ activation in microglia (1) and likely in astrocytes and neurons (not depicted). Possibly, PGE_2_ released from microglia binds its EP_1_ metabotropic receptors and produces the release of Ca^2+^ from intracellular stores (2). The increases in [Ca^2+^]_*i*_ is a known condition that evokes opening of Panx1 channels (Panx1 CHs), allowing the release of glutamate and ATP through them (3). ATP released via Panx1 CHs activates P2X_7_ receptors (4), triggering a self-perpetuating mechanism, in which high levels of [Ca^2+^]_*i*_ could reactivate Panx1 CHs in microglia. In addition, paracrine release of glutamate and ATP from microglia could act on neighboring or distant neurons, resulting in the activation of P2X_7_ (5) and NMDA (6) receptors, respectively. The latter enhances the activity of neuronal Panx1 CHs (7), allowing more release of ATP/glutamate and altering physiological functions of neurons (not depicted). Prenatal nicotine and postnatal high-fat/cholesterol diet could increase the release of glutamate and ATP from astrocytes via opening of Cx43 hemichannels (Cx43 HCs; 9). It is possible that microglia through the release of pro-inflammatory molecules (e.g., IL-1β and TNF-α) could contribute to the opening of astroglial Cx43 HCs via the activation of a p38MAPK/iNOS-dependent pathway.

Glutamate and ATP are considered key mediators on neuron-glia crosstalk. Thereby, their release through membrane proteins and vesicles is tightly regulated (Fields and Burnstock, 2006; Perea and Araque, 2010). In fact, high concentrations of glutamate and ATP at the synaptic cleft under pathological conditions could result in neurotoxicity (Lau and Tymianski, 2010; Arbeloa et al., 2012; Ashpole et al., 2013). As mentioned before, part of neuronal damage could depend on the release of glutamate and ATP via HCs and PCHs (Takeuchi et al., 2006; Garré et al., 2010; Orellana et al., 2011a,b). Both glutamate and ATP released by glial cells trigger the activation of neuronal NMDA and P2X7 receptors, which result in the opening of neuronal Panx1 channels and further cell death (Orellana et al., 2011a,b). Our results indicate that release of glutamate and ATP evoked by prenatal nicotine and postnatal HFC diet occurred via Cx43 and Panx1 unopposed channels, as it was inhibited by TAT-L2, Gap19, Gap26, ^10^panx1 and probenecid. Nevertheless, given that neuronal Etd uptake still persist under Cx43 but not Panx1 channel blockade, it seems that glutamate and ATP released from microglia rather than astrocytes are the major contributors to the opening of Panx1 unopposed channels in neurons (Figure 7).

How does the exposure to prenatal nicotine and postnatal HFC diet induce the opening of Cx43 and Panx1 unopposed channels? Previous studies have demonstrated that opening of these channels in microglia and astrocytes results on the activation of an iNOS/COX_2_/EP_1_ receptor- and p38MAPK/iNOS-dependent pathway, respectively (Retamal et al., 2007; Orellana et al., 2013). In agreement with that mechanism, Etd uptake and gliotransmitter release were nearly abolished by blockers of iNOS, COX_2_ and EP_1_ receptor, suggesting that activation of Cx43 and Panx1 unopposed channels likely occurred downstream of this pathway. Given that activation of EP_1_ receptors raises [Ca^2+^]_*i*_ (Woodward et al., 2011), opening of these channels possibly occurred by this mechanism, which is coherent with previous studies showing that increased levels of [Ca^2+^]_*i*_ are necessary for gliotransmitter release via HCs and PCHs (Locovei et al., 2006; Torres et al., 2012). This is also in agreement with the fact that P2X_7_ receptor activation, a well known mechanism that increases [Ca^2+^]_*i*_, was required to induce the release of glutamate and ATP we observed. By contrast, blockade of NMDA receptors, whose activation also enhances [Ca^2+^]_*i*_ levels, did not induce the same response. These data support the idea that ATP, but not glutamate, evokes its own release via Panx1 unopposed channels, and subsequent activation of purinergic receptors in microglia, as has been previously observed (Orellana et al., 2013; Figure 7). Previous studies have described that astrocytes exposed to activated microglia exhibit an increased Cx43 hemichannel opening sensitive to L-NAME (a broad range NOS inhibitor) and p38 MAPK inhibitors (Retamal et al., 2007). Therefore, it is conceivable to speculate that along with direct effect of nicotine and HFC diet on astrocytes, microglia might also contribute to the opening of astroglial HCs by releasing pro-inflammatory cytokines (see below; Figure 7). Whether specific crosstalk (e.g., through P2X_7_ receptors, HCs and PCHs) between astrocytes and microglia could explain their different contribution to neuronal Panx1 channel opening will be a matter of future investigation.

It has been described that nicotine exposure increases peripheral and brain levels of inflammatory cytokines, including IL-1β (Lau et al., 2006; Bradford et al., 2011). We speculate that prenatal nicotine exposure could affect the inflammatory state of dams, resulting in epigenetic modification of brain genes, leading to permanent changes in gene expression and long-term changes in structure and function (Boksa, 2010). Here, we found that the brain of adult offspring from nicotine-treated dams are sensitized to postnatal HFC diet, as has been previously described to occur with others environmental challenges (Slotkin et al., 1991; Bilbo et al., 2005). Given that feeding mice a cholesterol-enriched diet during adulthood results in a general inflammatory state (Thirumangalakudi et al., 2008; Lewis et al., 2010), this condition could act as a second inflammatory challenge affecting the CNS. Accordingly, we found that prenatal nicotine and postnatal cholesterol-enriched diet induced higher serum levels of IL-1β compared to control conditions.

Elevated blood levels of cytokines correlate with increased brain levels of cytokines (Erickson and Banks, 2011), being the latter closely linked to activation of iNOS, COX_2_ and EP_1_ receptors (Vinukonda et al., 2010; Sheng et al., 2011; Samy and Igwe, 2012). Therefore, it is plausible to speculate that increased brain levels of IL-1β and activation of iNOS/COX_2_/EP_1_ receptor pathway could be involved in the increased opening of Cx43 and Panx1 unopposed channels observed in our model (Figure 7). Supporting this idea, IL-1β causes opening of HCs and changes connexin expression in brain cells (Retamal et al., 2007; Froger et al., 2009, 2010; Orellana et al., 2011a; Xiong et al., 2012). Here, we observed that prenatal nicotine and postnatal HFC diet reduced Cx43 expression in astrocytes. Given that surface HCs account for ~11% of total Cx43 under resting conditions (Schalper et al., 2008), making them less detectable by immunofluorescence than gap junctions plaques, a reduction on Cx43 immunodetection not necessarily implicates a decrease on surface HCs or in their activity. On the other hand, Panx1 expression was increased in pyramidal neurons. It is possible that part of Etd uptake observed in pyramidal neurons could rely on this phenomenon. Further studies are required to elucidate whether changes in protein expression could contribute as well to the Cx43 and Panx1 unopposed channel activity triggered by prenatal nicotine and postnatal HFC diet.

Diverse studies have shown that cell and tissue responses to injuries depend on properties of the cells (e.g., age, hormonal exposure, and stage of cell cycle) and insult (e.g., duration, intensity, and quality). Moreover, CNS responses depend on interactions between their constituent cells, including chemical and electrical transmission as well as paracrine and autocrine signaling (e.g., by cytokines and ROS), possibly mediated by HCs and PCHs. In most chronic diseases, additional mechanisms are progressively added to the primary cause and thus, complicating the assignment of contribution of each factor to the final condition. Under this view, we speculate that the combined effect of two stressors (exposure to prenatal nicotine and postnatal HFC diet) resulted in our system in an synergic outcome on the increased activity of HCs and PCHs in brain cells, as has been previously observed in other inflammatory models (Orellana et al., 2010). Despite the difficulty of assigning contributions to connexin and pannexin unopposed channels in the pathogenesis of neurodegenerative diseases, recent studies using homo and/or heterocellular cultures have provided clues to elucidate this matter (Sáez and Leybaert, 2014). Although our model does not recapitulate completely mechanisms underlying brain abnormalities induced by maternal cigarette smoking, it allows us to dissect the specific contribution of HCs and PCHs expressed by individual brain cell types. Our findings bring new light on how gliotransmitters and the unbalance on paracrine signaling mediated by HCs and PCHs could contribute to developing brain abnormalities induced by different stressors during pregnancy.

## Conflict of interest statement

The authors declare that the research was conducted in the absence of any commercial or financial relationships that could be construed as a potential conflict of interest.
